# Physicochemical Properties and Structural Study of Heat Treatment-Modified Chinese Yam (*Dioscorea opposita* Thunb.) Starch–Ferulic Acid Complexes

**DOI:** 10.3390/foods14101761

**Published:** 2025-05-15

**Authors:** Sandu Xie, Yanping Lei, Huiqing Chen, Shuqi Liu, Xiaojuan Lin, Zebin Guo, Yi Zhang, Baodong Zheng

**Affiliations:** 1 Fujian Provincial Key Laboratory of Quality Science and Processing Technology in Special Starch, Fujian Agriculture and Forestry University, Fuzhou 350002, China; xsdfst@163.com (S.X.);; 2 School of Life Sciences and Chemistry, Minnan Science and Technology College, Quanzhou 362332, China; 3 College of Food Science, Fujian Agriculture and Forestry University, Fuzhou 350002, China

**Keywords:** Chinese yam starch, ferulic acid, complexation, structure, physicochemical property

## Abstract

In this study, complexes of pregelatinized Chinese yam starch with ferulic acid (PCYS+FA) were prepared using a boiling water bath, with varying levels of Chinese yam starch (CYS) and ferulic acid (FA). The investigation focused on the effects of FA addition (3%, 9%, and 15%) on the physicochemical properties and structure of PCYS+FA complexes. The solubility, swelling, and water-holding capacity of PCYS+FA were compared with those of CYS, with the solubility and swelling showing a gradual enhancement with increasing FA content. The incorporation of FA reduced the thermal stability of CYS, decreasing the initial degradation temperature from 245.94 °C (CYS) to 228.17 °C (PCYS+15%FA). Infrared spectroscopy revealed that CYS and FA were bound through non-covalent intramolecular hydrogen bonding. Furthermore, X-ray diffractograms showed that FA and CYS formed a V-type complex, in which the crystallinity of PCYS reached a minimum of 3.72%, and the degree of molecular ordering was reduced. Scanning electron microscopy analysis demonstrated that FA adhered to the surface of starch granules, resulting in the formation of pores that facilitated the entry of FA molecules into the internal crystal region of starch, allowing them to interact with starch molecules.

## 1. Introduction

Chinese yam (*Dioscorea opposita* Thunb.) is a subtropical root crop widely grown in China, and its tubers are a functional food with both edible and medicinal values [1]. Chinese yam is rich in carbohydrates, saponins, phenolics, starch, and proteins, offering various functions such as antidiabetic, anticancer, antioxidant, and immunomodulatory effects [2]. However, the high moisture content of fresh yam makes it difficult to preserve and time-consuming to handle. Thus, yam flour is usually made by drying and milling, serving as a base for processed products such as yam chips and yam cakes. Due to its high nutritional value, the development and utilization of Chinese yam starch (CYS) still has great potential.

CYS is prone to ageing and deterioration. During food manufacturing, the structure of starch granules is destroyed, exposing the constituent starch molecules [3]. Consequently, the water absorption and water-holding capacity of the starch are reduced, which is not conducive to its industrial application [4], thus it is imperative to enhance the functional properties of starch. Furthermore, its fast-digesting properties contribute to a high glycemic index, which can lead to rapid spikes and drops in blood glucose levels. These fluctuations may increase the risk of obesity and cardiovascular disease. Recent studies have shown that the physical blending of starch with phenolic acids can significantly enhance its pasting characteristics, rheological properties, and in vitro digestibility, thereby mitigating its adverse effects on blood glucose [5]. Carvalho et al. [6] suggested that the formation of phenolic compounds and starch complexes can promote the development of foods enriched with slow-digesting and resistant starch, providing safer starchy foods for diabetics. Polyphenol is a natural substance with a powerful antioxidant capacity that scavenges free radicals in the body and slows aging, among other properties. The hydrophobic interactions and hydrogen bonding resulting from the binding of polyphenols to starch greatly reduce digestibility and increase the content of resistant starch. This modification method can enhance not only the microstructure of starch but also improve its stability, water-holding, and antioxidant properties.

Ferulic acid (FA) is a naturally occurring phenolic acid with a wide range of biological activities, including antioxidant, anti-inflammatory, antimicrobial, anticancer, and hypolipidemic effects. The interaction between starch and phenolic compounds has been shown to enhance starch stability, thereby improving the functional properties of starch and paving the way for its application in the functional food industry [7]. The abundance of hydroxyl groups in its structure facilitates electrostatic, hydrogen bonding, and van der Waals interactions with starch, resulting in the formation of complexes [8]. The addition of different concentrations of FA to 3D-printed rice starch gels effectively reduced the crystallinity and short-range ordering [9]. This finding suggests that FA may possess the capacity to enhance the structure of starch, which could inform the development of healthier products. Song et al. [10] demonstrated that the application of high hydrostatic pressure (HHP) can facilitate the formation of corn starch and FA complexes. HHP improved the digestibility of the corn starch–FA complex by increasing the proportion of slowly digestible and resistant starch. Maibam et al. [11] found that the solubility index of pregelatinized Euryale ferox kernel starch was 8.32%. The solubility index of the solution with 10% ferulic acid was 16.36%. The incorporation of ferulic acid improved starch–water interactions and increased the water solubility of ferulic acid–starch complexes. In recent years, there has been a growing interest in leveraging food processing technologies to manipulate the interactions between polyphenolic compounds and starch. This approach can significantly influence the functional properties and structural properties of starch, offering potential benefits for health and food functionality [12].

FA affects the properties and structure of natural starches to different degrees. Therefore, in this experiment, complexes of pregelatinized CYS were prepared with varying concentrations of FA (PCYS+FA). The effects of FA addition on the physicochemical properties of PCYS were studied, and the changes in its microstructure were further investigated using Fourier infrared spectroscopy and X-ray diffractometry. These findings will help to elucidate the mechanism of polyphenol–starch interactions under heat treatment, providing a theoretical basis for improving functional starch products and developing new healthy foods.

## 2. Materials and Methods

### 2.1. Materials

Chinese yam (*Dioscorea opposita* Thunb.) starch (food grade, 69.72 ± 0.50% amylopectin content, 26.28 ± 0.91% amylose content) was obtained from Fujian Shange Agricultural Comprehensive Development Co., Ltd. (Quanzhou, China). FA (99% purity) was purchased from Shanghai Macklin Biochemical Co., Ltd. (Shanghai, China). The experimental water was treated with a water purification machine (MR-01593-50G, Midea Group Co., Ltd., Foshan, China) to obtain deionized water (pH = 7.0 ± 0.1). All chemicals used were of analytical grade.

### 2.2. Sample Preparation

Fresh Chinese yam tubers, following removal of the unusable parts, were mixed with pure water to create a slurry. The fresh Chinese yam slurry was then washed with twice the volume of pure water and anhydrous ethanol to clarify the mixture, yielding a top layer of clear liquid. The sediment obtained using this method was designated as CYS. The CYS was dried in an oven (101-1AB electric blast drying oven, Tianjin, China) at 40 °C to a constant weight and filtered through a 100-mesh screen.

### 2.3. Preparation of Chinese Yam Starch–Ferulic Acid Complex

The CYS-FA complexes were produced according to previous methods with some modifications [13]. CYS and deionized water were mixed (5%, g/mL) with continuous agitation and heated at 100 °C for 10 min. Then, FA was added at ratios of 3%, 9%, and 15% (*w*/*w*) per 100 g of CYS. The mixture was continually heated at 80 °C for 20 min. Subsequently, the samples were washed with an absolute ethyl alcohol solution and centrifuged at 4000 rpm for 10 min. The mixture was dried at 40 °C for 24 h, pulverized, and sieved through a 100-mesh screen. PCYS was prepared according to the same procedure without FA. The complexes containing 3%, 9%, and 15% FA were labeled as PCYS+3%FA, PCYS+9%FA, and PCYS+15%FA, respectively.

### 2.4. Determination of Swelling Power and Solubility

The effect of FA on the swelling power and solubility of PCYS was determined in accordance with the methodology outlined by Xu et al. [14] with modifications. First, 100 mg of the CYS sample (*W*_1_) was measured, combined with 5 mL of distilled water, and added to a dry centrifuge tube. The tube was placed in a constant-temperature water bath at 50, 60, 70, 80, and 90 °C for 20 min, with the temperature recorded every 5 min. Following this, the tube was removed from the water bath and cooled to room temperature. The tube was then centrifuged at 6000 rpm for 15 min. The resulting upper layer was decanted into a weighing bottle and dried in an oven at 105 °C until a constant weight was reached (*W*_2_). The weight of the residue (*W*_3_) was then determined, and the solubility and swelling power were calculated according to the following formula:

$$S(\%)=\frac{W_{2}}{W_{1}}\times 100\;SP(g/g)=\frac{W_{3}}{W_{1}(100-S)}$$

### 2.5. Determination of Water Holding Capacity (WHC)

The water-holding properties of starch were analyzed using the method described by Sun et al. [15] with modifications. First, 100 mg of the starch sample (*W*_0_) was transferred to a dry test tube and weighed (*W*_1_). Then, 2 mL of distilled water was added, and the tube was heated for 15 min at 50, 60, 70, 80, and 90 °C. The tube was cooled to room temperature and centrifuged for 20 min at 6000× *g*. After pouring off the supernatant, both the centrifuge tube and precipitate were weighed (*W*_2_). The WHC of the sample was calculated using the following equation:

$$\mathrm{WHC}(\%)=\frac{W_{1}-W_{2}}{W_{0}}\times 100$$

### 2.6. X-Ray Diffraction (XRD) Analysis

Following the method described by Wang et al. [16], with a slight modification, an appropriate amount of the sample to be tested was spread on a rectangular aluminum sheet and placed into an X-ray diffractometer (Rigaku Corporation, Tokyo, Japan) for measurement using Cu-Kɑ radiation. The following conditions were applied: tube pressure, 40 kV; tube current, 30 mA; scanning area, 3–45°; scanning speed, 2°/min; sampling step width, 0.02°; and scanning mode, continuous.

### 2.7. Fourier Transform Infrared Spectroscopy (FTIR) Analysis

To obtain the infrared spectra of the samples, FT-IR analysis (IR Affinity-1S, Shimadzu Enterprise Management, Shanghai, China) was performed according to the method described by Jiang et al. [17]. A specific amount of the sample was dried to a constant weight and combined with potassium bromide (with a weight ratio of 1:100) in an agate bowl. The mixed sample was ground until no particles were visible to the naked eye, spread evenly in a film press, and pressed into a tablet. For FT-IR analysis, the scanning range was 4000–400 cm^−1^, with 32 accumulations and 4 cm^−1^ resolution.

### 2.8. Thermal Properties Analysis

Briefly, 10.0 mg of the starch sample was carefully weighed and evenly flattened in a platinum crucible. Thermogravimetric analysis was subsequently performed using a thermogravimetric analyzer (SDT Q600, TA Instruments, Newcastle, DE, USA) under a nitrogen atmosphere, with a flow rate of 60 mL/min, a temperature ramp rate of 10 °C/min, and a temperature range of 25–600 °C.

### 2.9. Scanning Electron Microscopy (SEM) Analysis

SEM observations of the PCYS starch complex formed from CYS and varying amounts of FA were performed according to the method described by Li et al. [18]. A small amount of starch was uniformly sprinkled onto a double-sided adhesive tape attached to a circular sample holder. The sample was then sprayed with a gold layer using a Baltzers SCD 004 sputtering apparatus to enhance its conductivity. Finally, the sample was observed and imaged at 1000× and 10,000× magnifications using a scanning electron microscope (TESCAN MIRA, TESCAN Group a.s., Brno, Czech Republic).

### 2.10. Statistical Analysis

All experiments were repeated at least three times, and the results are presented as mean ± standard deviation. A one-way analysis of variance was performed using SPSS 27.0 software, employing Duncan’s multiple range test for post hoc statistical analysis (*p* < 0.05) to assess significant differences between the groups. Further graphing was performed using the Origin 2021 software.

## 3. Results and Discussion

### 3.1. Solubility and Swelling Power

The solubility and swelling power of starch are based on its ability to dissolve and swell during the pasting process. These were calculated based on the degree of interaction between the starch chains in the amorphous and crystalline regions of starch granules [19]. The water solubility index and swelling power of the PCYS+FA complexes—containing different amounts of FA—are presented in Table 1 and Table 2. There was a correlation between the amount of FA added and the water solubility index of CYS. The solubility of starch showed a clear positive correlation with increasing temperature. As the temperature rose, the starch particles absorbed water and expanded, enabling the release of amylose and small amylopectin, thereby gradually increasing the solubility of the starch. The increase in swelling force and solubility can be attributed to the melting of starch crystals. The transition of starch chains from spiral to coil led to an increase in enthalpy, which negated the effects of hydrogen bonding in the crystallization zone [3]. The water solubility index of PCYS increased from 9.00% to 10.67% when the FA addition was increased to 15% at 70 °C. Furthermore, the water solubility index of PCYS+15%FA exhibited a more substantial increase at 90 °C. The solubility of the substance was found to be 68.70% higher than that of PCYS. As the temperature increased, the amorphous zone became more permeable to water molecules, thereby disrupting the hydrogen bonds within it [20]. This process facilitated the formation of new hydrogen bonds between the starch and water molecules, enhancing the solubility of starch. This finding suggests a direct correlation between elevated temperature and enhanced starch solubility, a process that contributes to the enhancement of functional properties [21]. The solubility of PCYS+15%FA was higher than that of other starches at all temperatures. The increase in the internal interstitial space within the complex granules at this level of FA addition, along with the enhanced hydrogen bonding interactions between the phenolic hydroxyl groups in FA and the starch granules, improved starch solubilization [22]. Karunaratne et al. [23] found that the water solubility index of maize starch increased from 9.4% to 21.0% at 20% FA, concluding that the addition of FA lowered the pH of the system and increased the solubility.

The swelling degree of the PCYS+FA complexes exhibited a gradual increase in response to elevated temperatures. At temperatures of 50, 60, and 70 °C, the solubility of the starch increased more gradually. However, the swelling degree reached a maximum value at 90 °C. The increased swelling power of starch enabled greater water absorption and a fluffier structure during processing, improving the texture and flavor of the food. As the temperature gradually approached the pasting temperature of starch, the polar groups in the starch were exposed and interacted with the water molecules. This caused the starch granules to rapidly absorb the surrounding water, resulting in noticeable swelling. Consequently, water molecules and FA were more likely to enter the interior of the starch granules, leading to a gradual enlargement of the starch granule interstices [24]. And the starch swelling power showed an almost positive correlation with the increase in ferulic acid addition. The addition of FA can increase the swelling power of PCYS, which may be attributed to its high content of straight-chain starch. This straight-chain starch interacts with the dispersed FA, reducing interconnections between the starch chains and increasing the swelling capacity of starch granules during the pasting process.

### 3.2. Water-Holding Capacity

During food manufacturing, the WHC of starch directly influences the texture, flavor, and mouthfeel of the end product. Thus, the effect of varying FA concentrations on the water-holding capability of PCYS complexes was examined (Table 3). The findings indicated an enhancement in the water-holding capacity of starch with an increase in temperature from 50 to 90 °C, which was consistent with the results observed for solubility and swelling power. The starch granules expanded as the temperature increased, resulting in an increased WHC. The water-holding capacity of PCYS was considerably higher than that of CYS, indicating that starch damage resulted in a certain degree of starch gelatinization during the heating process. The highest WHC was demonstrated by PCYS+3%FA at a temperature of 90 °C. Following the addition of FA, the compound entered the interior of the starch granules through hydrothermal treatment and interacted with the non-helical branched-chain starch molecules through intermolecular hydrogen bonding [25]. This process attenuated the hydrogen bonding of the starch molecules, resulting in a looser structure that enabled water molecules to penetrate the starch granules with ease. The hydroxyl group in FA exhibited hydrophilic polarity, allowing it to interact with water molecules and enhance the WHC of the starch. Starch gelatinization refers to the physical and chemical transformations that occur when starch is heated in the presence of water. As the temperature rises, starch granules absorb water and swell initially. Upon reaching the gelatinization temperature range (e.g., 90 °C for CYS), the ordered crystalline structure of starch is disrupted, hydrogen bonds break, and molecular chains disassociate into an amorphous network. This structural reorganization significantly enhances water-holding capacity (WHC) by enabling starch to trap more free water molecules via hydrogen bonding and capillary forces [26]. For pre-gelatinized starch (PCYS), which already exhibits partial gelatinization damage, a gelatinization-like process can occur at lower temperatures (50–90 °C), leading to a pronounced increase in WHC with rising temperature. Post-gelatinization, the formation of an open porous network structure further improves water migration freedom, thereby optimizing the WHC [27]. Consequently, the PCYS+FA complex exhibited a notable water-holding capacity. The water-holding property of CYS significantly increased at 90 °C, coinciding with its gelatinization temperature. This enhancement was attributed to the interaction of polar groups within the starch molecules with water, resulting in the substantial augmentation of their WHC. When FA was added to the CYS system and hydrothermally treated, it formed intermolecular hydrogen bonds with the starch molecules via carboxyl and hydroxyl groups [28]. Lower FA concentrations led to limited starch–FA binding, a minor impact on starch–starch hydrogen bonds, and negligible improvement in the WHC. At 3% FA, enhanced penetration into starch granules disrupted hydrogen bonds within non-helical amorphous regions of amylopectin, loosened the structure of the starch, and significantly boosted the WHC. But higher FA concentrations can cause FA aggregation, reducing interaction sites or altering the complex structure, which can stop or even decrease the WHC. Hence, in the PCYS+FA complex, 3% FA provides the best water-holding capacity due to optimized interaction and structure [28]. The PCYS+FA complexes exhibited an optimal water-holding capacity, suggesting their potential application as effective thickeners in food processing.

### 3.3. Long-Range Ordered Structure

**Figure 1:**
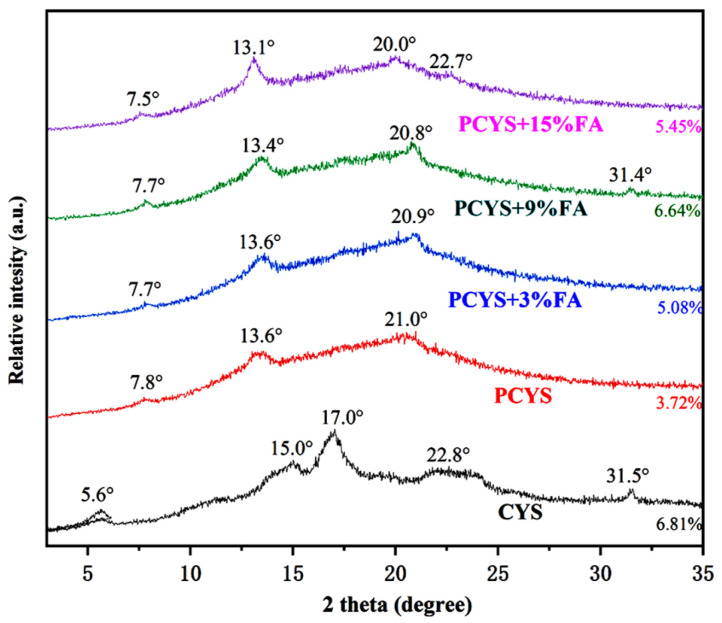
X-ray diffraction patterns of CYS combined with FA of different concentration. CYS: Chinese yam starch, PCYS: Pregelatinized Chinese yam starch, PCYS+3%FA: Pregelatinized Chinese yam starch with 3% ferulic acid, PCYS+9%FA: Pregelatinized Chinese yam starch with 9% ferulic acid, PCYS+15%FA: Pregelatinized Chinese yam starch with 15% ferulic acid.

The effects of FA addition on the long-range ordered structure of CYS were explored using XRD. The crystallographic pattern of CYS showed a typical B-type structure, with strong diffraction peaks at 2 theta = 5.6°, 5.0°, 17.0°, and 22.8° (Figure 1) [29]. The diffraction peaks of PCYS samples at 15.0°, 17.0°, and 22.8°, as well as the B-type crystalline features, gradually disappeared, indicating that pregelatinization destroyed the CYS helical structure. The PCYS samples with FA added showed a weak peak at 20°, with PCYS-15% FA showing characteristic peaks at 2 theta = 7.5°, 13.1°, 20.0°, and 22.7°, indicating that the starch formed a V-type inclusion complex with FA [30]. This phenomenon was primarily attributable to hydrogen bonding and hydrophobic interactions between FA and PCYS, which impeded the formation of A-type crystals and concurrently promoted the formation of V-type crystals [31]. The intensity of the characteristic peaks near 13.0° increased significantly with increasing FA content, demonstrating the significant effect of FA on the crystal properties of PCYS-FA.

The extent of the effects of FA on the crystal properties is directly proportional to the amount of acid added. During starch extrusion, phenolics are more likely to enter the helical cavity of amylose and form V-complexes [32]. As shown in Figure 1, the relative crystallinity of CYS decreased from 6.81% to 3.72% following pasteurization, suggesting that the pasting treatment disrupted the long-range ordered structure of the starch. In contrast, the relative crystallinity of PCYS+FA was higher than that of PCYS, with the relative crystallinity of PCYS+9%FA reaching 6.44%. This result suggests that excess FA may compete with CYS for water molecules during the pasting process. The FA molecules interacted with the starch chains, reducing the swelling of the starch granules and promoting repolymerization between the starch chains [33]. Li et al. [34] observed that starch chains can bind with the hydroxyl groups of polyphenols, enhancing the long-range ordering of starch molecules and resulting in a more compact crystalline region. However, the presence of FA diffraction peaks may also be responsible for the increased relative crystallinity [9].

### 3.4. Short-Range Ordered Structure

**Figure 2:**
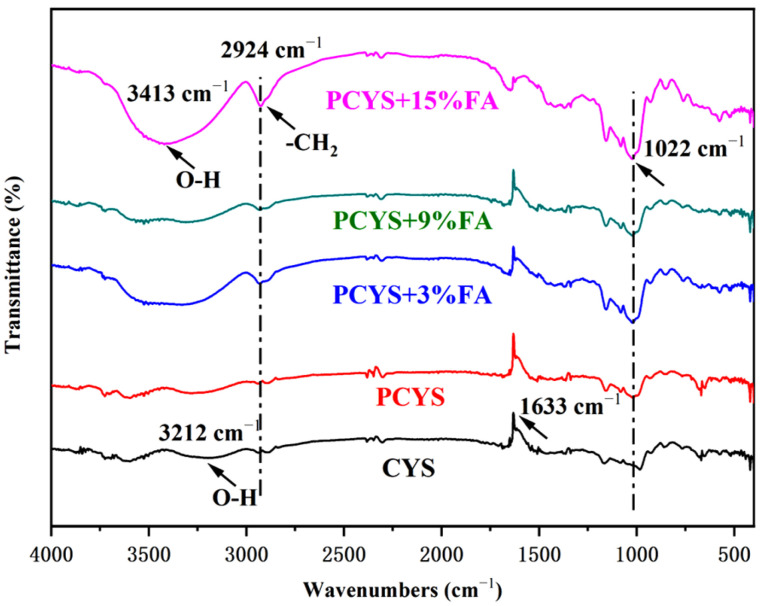
FT−IR spectra of CYS combined with FA of different concentration. CYS: Chinese yam starch, PCYS: Pregelatinized Chinese yam starch, PCYS+3%FA: Pregelatinized Chinese yam starch with 3% ferulic acid, PCYS+9%FA: Pregelatinized Chinese yam starch with 9% ferulic acid, PCYS+15%FA: Pregelatinized Chinese yam starch with 15% ferulic acid.

FT-IR analysis was used to further investigate the molecular conformation and changes in the bonding energy of chemical groups in the complex formed by CYS and FA (Figure 2). The starch fingerprint region associated with carbohydrates exhibited a spectral range of 1200–800 cm^–1^. This phenomenon indicated alterations in the short-range structure of the C–C and C–O bonds during the stretching process. The characteristic starch absorption band due to O–H stretching appeared at 3212 cm^–1^, along with a characteristic C–H stretching vibration peak at 2924 cm^–1^. When FA was added to the CYS sample, the O–H stretching vibration peak of PCYS+15%FA shifted to 3413 cm^–1^. The broad absorption peaks in this region are indicative of starch stretching vibrations with typical intermolecular associations. As the FA concentration increased, the CYS absorption band near 3500–3000 cm^–1^ shifted to lower wave numbers, indicating strengthened hydrogen bonding interactions between FA and the starch molecules. This finding is in accordance with the observations reported by Meng et al. [35], who found that the starch formed non-covalent bonds—including hydrophobic interactions and hydrogen bonds—with FA during gelatinization. Moreover, the hydrogen bonding resulting from the interactions between starch and phenolics was stronger than the intermolecular hydrogen bonding present in the starch chains [11]. An absorption peak characteristic of the ester bond (C=O) appeared near 1633 cm^–1^. Raza et al. [36] confirmed the presence of –C=O bond stretching and vibrational absorption peaks at 1645 cm^–1^ for the rice starch–chlorogenic acid complex. FA is a phenolic acid with the structure of cinnamic acid. As the molecular structure of FA contains carboxyl groups, while that of starch contains hydroxyl groups, the characteristic peak observed indicates the compounding of starch and FA. When the FA molecules entered the hydrophobic cavity of the starch, the polar groups were further exposed to the hydrophobic environment. As a result, the intensity of the absorption peaks became weaker with the increase in FA content [37].

The peak at 1022 cm^–1^ corresponds to C–O absorption vibration, while the peak at 1022 cm^−1^ can be ascribed to the amorphous structure of starch. The ratio of ordered to amorphous structures in starch can be expressed by calculating the intensity ratio of the peaks at 1047/1022 [17]. The magnitude of this ratio indicates the degree of order within the starch granule, with larger ratios indicating a higher degree of order. As demonstrated in Table 4, the *R*_1047/1022_ value of starch was lowest in its natural state, while the *R*_1047/1022_ value of PCYS increased after heating and pasting. In contrast, the *R*_1047/1022_ value of PCYS, with the addition of 3% FA, reached a maximum but decreased with the addition of 15% FA. The starch gel network structure was more ordered with low concentrations of FA but became looser and less ordered with high concentrations of FA. The *R*_995/1022_ value is a reliable indicator of the extent of starch double helix formation, which increased following starch pregelatinization. The PCYS structure was also more stable after the addition of FA, suggesting the potential of FA to slow the dissociation of branched starch.

### 3.5. Thermal Properties

**Figure 3:**
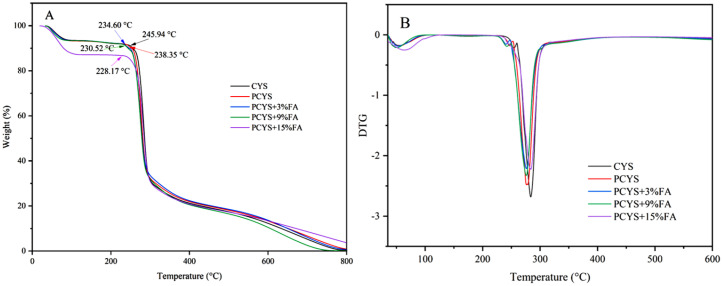
Thermogravimetric curves (**A**) and corresponding derivative curves (**B**) of CYS−FA complexes. CYS: Chinese yam starch, PCYS: Pregelatinized Chinese yam starch, PCYS+3%FA: Pregelatinized Chinese yam starch with 3% ferulic acid, PCYS+9%FA: Pregelatinized Chinese yam starch with 9% ferulic acid, PCYS+15%FA: Pregelatinized Chinese yam starch with 15% ferulic acid.

The effects of different FA concentrations on the thermal stability of CYS at elevated temperatures were elucidated from the thermogravimetric results. The thermogravimetric analysis plots and corresponding derivative curves of natural CYS and the complexes formed with different FA additions are shown in Figure 3. Both natural starch and the PCYS-FA complexes showed two similar phases of thermal decomposition. The initial mass loss of 15%, occurring between 30 and 100 °C, was primarily due to the evaporation of water from the starch samples. The second mass loss, occurring between 200 and 400 °C, was mainly attributed to the thermal degradation of starch. The initial degradation temperatures of CYS and PCYS were 245.94 and 238.35 °C, respectively. This phenomenon was primarily attributed to the decomposition of starch molecular chains during the heating of CYS. The initial degradation temperature of PCYS decreased with increasing FA content, with the lowest temperature observed for PCYS+15%FA (228.17 °C). The incorporation of FA into the system influenced the thermodynamics of PCYS, concomitantly reducing the thermal stability of CYS. The reduced thermal stability of the PCYS+FA compound suggests that it may be capable of undergoing specific chemical reactions at lower temperatures. This property may be relevant to its potential application in environmentally friendly packaging materials, where degradability is a key consideration. Wang et al. [16] found that the initial decomposition temperature of the chlorogenic acid–lotus starch complex was lower than that of lotus starch under autoclave treatment conditions. The addition of chlorogenic acid disrupted the ordered structure of the lotus starch, reducing its thermodynamic stability. As can be seen from the DTG curves in Figure 3B, the decomposition temperatures at the maximum thermal decomposition rate of PCYS and the PCYS-FA complexes were slightly lower than that of CYS, suggesting a lower thermal stability of the FA–starch complexes. This finding further confirms that the thermodynamic stability of FA decreased to some extent after complexation with CYS at varying addition levels. Furthermore, Mao et al. [38] found that the ordered structure of potato starch gels was disrupted by the addition of phenolic acid, leading to reduced thermodynamic and rheological properties of the complexes.

### 3.6. SEM Analysis

**Figure 4:**
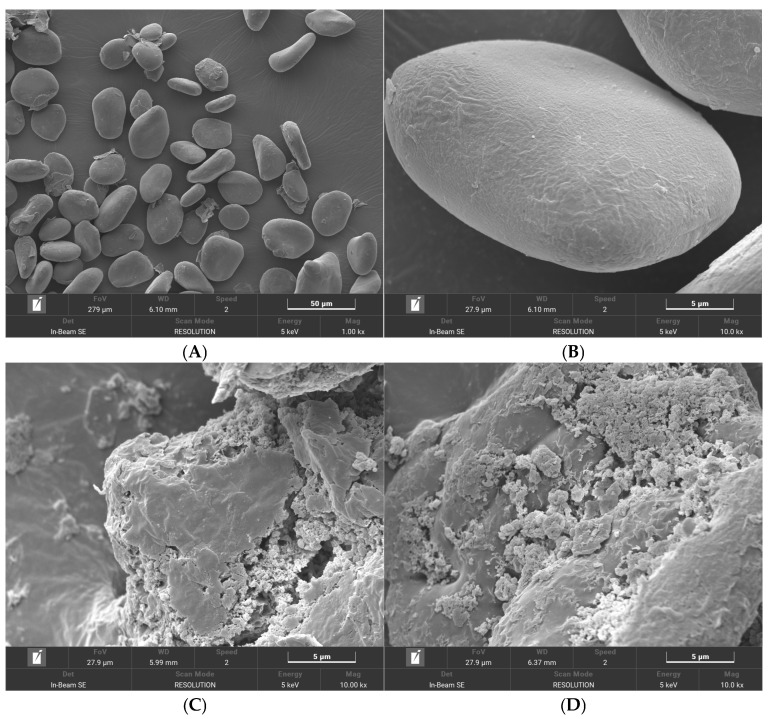

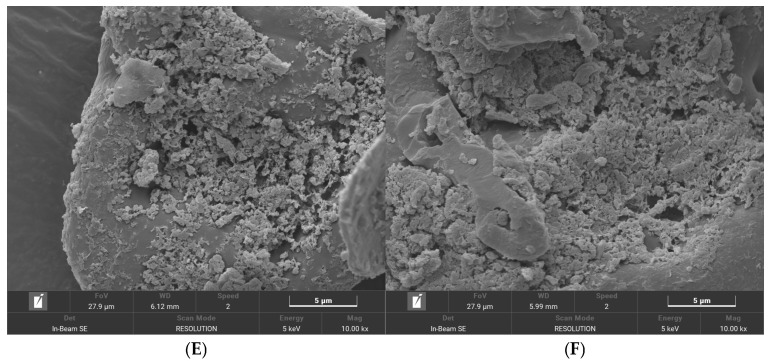
Changes in microstructure of CYS−FA complexes. (**A**) CYS (1.00 k× magnification); (**B**) CYS (10.00 k× magnification); (**C**) PCYS (10.00 k× magnification); (**D**) PCYS+3%FA (10.00 k× magnification); (**E**) PCYS+9%FA (10.00 k× magnification); (**F**) PCYS+15%FA (10.00 k× magnification). CYS: Chinese yam starch, PCYS: Pregelatinized Chinese yam starch, PCYS+3%FA: Pregelatinized Chinese yam starch with 3% ferulic acid, PCYS+9%FA: Pregelatinized Chinese yam starch with 9% ferulic acid, PCYS+15%FA: Pregelatinized Chinese yam starch with 15% ferulic acid.

The present study investigated the effects of FA addition on the morphology and structure of CYS granules using SEM. The surface of native CYS granules was smooth and devoid of holes, adopting circular or elliptical forms (Figure 4A,B). However, these granules displayed significant variations in size. Following the gelatinization of CYS, the starch particles expanded and ruptured. This process resulted in the formation of irregular clumps, which disrupted the morphology of the starch particles (Figure 4C). The pasting process thus resulted in a dissociation of the double helix starch structure and a disruption in the crystal structure. FA particles were observed to be scattered on the starch surface, indicating that FA did not disrupt the granular morphology of CYS (Figure 4D–F) [39]. The formation of pores on the starch granule surface was more visible when the FA content increased, indicating that FA promoted the formation of pores in the complex [40]. FA particles were dispersed on the surface when the FA concentration was 3%. Increasing the FA concentration to 9% caused an increase in the number of holes formed, with larger holes appearing at 15% FA. The strong hydrophobicity of the FA molecule can lead to the formation of cavities in the helical structure of straight-chain starch during the pasting process, driven by temperature and swelling pressure [41]. In this study, the addition of FA did not cause significant damage to the natural CYS granules. Instead, FA attached to the surface of the starch granules, resulting in the formation of pores. As a result, the FA molecules were more likely to enter the internal crystal region of the starch and interact with starch molecules.

## 4. Conclusions

In the present study, the effect of varying concentrations of FA on the physicochemical and structural characteristics of PCYS was examined. The swelling power, solubility, and WHC increased with rising temperature. Furthermore, the water solubility index of PCYS increased from 9.00% to 10.67% at 70 °C with 15% FA. The incorporation of FA influenced the thermodynamics of PCYS, causing an initial decrease in the degradation temperature that became more pronounced at higher FA concentrations. Additionally, FA interacted predominantly through non-covalent bonding and did not disrupt the granular morphology of the yam starch. This was evidenced by the SEM images, which revealed FA particles distributed on the starch surface. The findings of this study suggest that the incorporation of ferulic acid enhances the thermal stability and physical characteristics of pregelatinized Chinese yam starch complexes, thereby providing a theoretical foundation and a novel approach for the development of functional foods that address contemporary nutritional and health requirements.

## Figures and Tables

**Table 1:** Water solubility index of CYS in presence of FA.

Sample	Water Solubility Index (%)				
	50 °C	60 °C	70 °C	80 °C	90 °C
CYS	1.67 ± 0.58 ^c^	2.00 ± 1.00 ^b^	2.00 ± 0.00 ^c^	13.67 ± 5.51 ^a^	14.67 ± 4.73 ^a^
PCYS	5.33 ± 2.31 ^b^	6.67 ± 1.15 ^a^	9.00 ± 2.00 ^ab^	8.67 ± 1.53 ^a^	10.67 ± 5.51 ^a^
PCYS+3%FA	5.67 ± 1.15 ^b^	5.67 ± 2.31 ^a^	7.33 ± 1.53 ^b^	11.00 ± 2.00 ^a^	11.33 ± 2.52 ^a^
PCYS+9%FA	7.00 ± 2.00 ^ab^	7.00 ± 1.00 ^a^	8.00 ± 1.00 ^ab^	13.33 ± 0.58 ^a^	15.33 ± 8.39 ^a^
PCYS+15%FA	10.00 ± 2.00 ^a^	8.33 ± 2.08 ^a^	10.67 ± 2.08 ^a^	14.33 ± 2.31 ^a^	18.00 ± 7.81 ^a^

Values are presented as mean ± standard deviation. Different superscript letters within the same column indicate significant differences (*p* < 0.05). CYS: Chinese yam starch, PCYS: Pregelatinized Chinese yam starch, PCYS+3%FA: Pregelatinized Chinese yam starch with 3% ferulic acid, PCYS+9%FA: Pregelatinized Chinese yam starch with 9% ferulic acid, PCYS+15%FA: Pregelatinized Chinese yam starch with 15% ferulic acid.

**Table 2:** Swelling power of CYS in presence of FA.

Sample	Swelling Power (g/g)				
	50 °C	60 °C	70 °C	80 °C	90 °C
CYS	2.92 ± 0.17 ^c^	3.46 ± 0.20 ^b^	3.60 ± 1.16 ^b^	17.22 ± 2.31 ^b^	24.26 ± 2.57 ^a^
PCYS	13.96 ± 2.22 ^b^	19.70 ± 1.31 ^a^	22.29 ± 1.24 ^a^	22.70 ± 2.28 ^a^	25.60 ± 2.04 ^a^
PCYS+3%FA	15.05 ± 2.40 ^ab^	19.01 ± 2.19 ^a^	22.64 ± 1.60 ^a^	24.05 ± 0.72 ^a^	25.60 ± 0.43 ^a^
PCYS+9%FA	16.29 ± 2.03 ^ab^	18.95 ± 1.66 ^a^	21.30 ± 0.34 ^a^	23.83 ± 0.98 ^a^	26.38 ± 3.32 ^a^
PCYS+15%FA	17.76 ± 2.32 ^a^	19.53 ± 0.59 ^a^	22.31 ± 1.48 ^a^	24.1 ± 1.28 ^a^	26.49 ± 2.89 ^a^

Values are presented as mean ± standard deviation. Different superscript letters within the same column indicate significant differences (*p* < 0.05). CYS: Chinese yam starch, PCYS: Pregelatinized Chinese yam starch, PCYS+3%FA: Pregelatinized Chinese yam starch with 3% ferulic acid, PCYS+9%FA: Pregelatinized Chinese yam starch with 9% ferulic acid, PCYS+15%FA: Pregelatinized Chinese yam starch with 15% ferulic acid.

**Table 3:** Water-holding capacity of CYS in presence of FA.

Sample	Water-Holding Capacity (%)				
	50 °C	60 °C	70 °C	80 °C	90 °C
CYS	139.67 ± 26.27 ^c^	143.33 ± 14.98 ^b^	177.33 ± 48.19 ^c^	217.67 ± 2.31 ^b^	1073.67 ± 53.72 ^c^
PCYS	1144.67 ± 101.44 ^ab^	1322.00 ± 273.41 ^a^	1404.33 ± 100.01 ^ab^	1518.00 ± 91.79 ^a^	1631.33 ± 18.04 ^ab^
PCYS+3%FA	1111.00 ± 37.24 ^b^	1256.67 ± 93.39 ^a^	1544.00 ± 118.29 ^a^	1483.00 ± 177.79 ^a^	1710.67 ± 86.6 ^a^
PCYS+9%FA	1205.33 ± 112.52 ^ab^	1410.00 ± 182.05 ^a^	1461.67 ± 98.15 ^ab^	1574.33 ± 55.77 ^a^	1556.00 ± 37.00 ^b^
PCYS+15%FA	1261.67 ± 19.73 ^a^	1365.33 ± 125.09 ^a^	1367.33 ± 77.8 ^b^	1453.67 ± 56.54 ^a^	1545.33 ± 66.53 ^b^

Values are presented as mean ± standard deviation. Different superscript letters within the same column indicate significant differences (*p* < 0.05). CYS: Chinese yam starch, PCYS: Pregelatinized Chinese yam starch, PCYS+3%FA: Pregelatinized Chinese yam starch with 3% ferulic acid, PCYS+9%FA: Pregelatinized Chinese yam starch with 9% ferulic acid, PCYS+15%FA: Pregelatinized Chinese yam starch with 15% ferulic acid.

**Table 4:** Short-range ordered structure of composites of CYS and FA with different concentrations.

Samples	*R*_1047/1022_	*R*_995/1022_
CYS	1.009	0.984
PCYS	1.021	1.014
PCYS+3%FA	1.057	1.052
PCYS+9%FA	1.031	1.030
PCYS+15%FA	1.031	1.107

CYS: Chinese yam starch, PCYS: Pregelatinized Chinese yam starch, PCYS+3%FA: Pregelatinized Chinese yam starch with 3% ferulic acid, PCYS+9%FA: Pregelatinized Chinese yam starch with 9% ferulic acid, PCYS+15%FA: Pregelatinized Chinese yam starch with 15% ferulic acid.

## Data Availability

The original contributions presented in this study are included in the article. Further inquiries can be directed to the corresponding author.
